# Effects and mechanisms of phytohormones in enhancing the resistance of *Oocystis borgei* to high ammonium nitrogen stress

**DOI:** 10.1038/s41598-025-10398-z

**Published:** 2025-07-09

**Authors:** Xinyue Song, Yang Liu, Chengcheng Deng, Zhongdian Dong, Zhangxi Hu, Xianghu Huang, Changling Li, Ning Zhang

**Affiliations:** 1 Lab of Algae Resource Development and Aquaculture Environment Ecological Restoration, Fisheries College, Guangdong Ocean University, Zhanjiang, 524088 China; 2 Fisheries Research Institute, Sichuan Academy of Agricultural Sciences, Chengdu, 611731 Sichuan China; 3 Key Laboratory of Aquaculture in South China Sea for Aquatic Economic Animal of Guangdong Higher Education Institutes, Fisheries College, Guangdong Ocean University, Zhanjiang, 524088 China

**Keywords:** High ammonium nitrogen stress (HANS), Phytohormones, *Oocystis borgei*, Photosynthetic efficiency, Antioxidant capacity, Nitrogen metabolism, Ecology, Ecology

## Abstract

Excessive ammonium nitrogen (NH_4_^+^-N) in aquatic environments poses significant challenges for wastewater treatment and microalgae-based bioremediation. This study investigated the effects of phytohormones on enhancing the tolerance of *Oocystis borgei* to high NH_4_^+^-N stress and explored the underlying mechanisms. Growth of *O. borgei* was significantly inhibited by NH_4_^+^-N in a dose-dependent manner, with 500 mg·L^−1^ NH_4_^+^-N selected as the stress concentration. Supplementation with 10^−6^ M indole-3-acetic acid (IAA), gibberellic acid (GA3), or zeatin (ZT) significantly alleviated growth inhibition under high NH_4_^+^-N stress, increasing cell density by 22–26% and specific growth rate by 217–282% compared to the HAN group. No significant differences were observed among the tested phytohormones. Phytohormone treatments mitigated photosynthetic inhibition by enhancing chlorophyll a (Chla) and carotenoid (Car) contents, as well as Fv/Fm and φPSII values. While *rbcL* gene expression remained suppressed, phytohormones reduced oxidative stress by decreasing malondialdehyde (MDA) content and modulating superoxide dismutase (SOD) and catalase (CAT) activities. Additionally, phytohormones promoted nitrogen metabolism by significantly increasing glutamine synthetase (GS) activity and gene expression. This study provides insights into the protective mechanisms of phytohormones against high NH_4_^+^-N stress in *O. borgei*, offering potential strategies for enhancing algal-based treatment of high-ammonium wastewater.

## Introduction

Ammonia nitrogen is one of the most pressing pollutants in aquatic environments, posing severe threats to ecosystem health and human well-being due to its high toxicity and widespread presence in surface water systems^1–3^. Ammonia nitrogen in water predominantly exists in two forms: ionized ammonium (NH₄⁺) and unionized ammonia (NH₃). The relative concentrations of these forms are influenced by pH and temperature. At higher temperatures and pH values (e.g., > 30 °C and pH > 8.5), unionized ammonia predominates, while at lower temperatures and pH levels, ionized ammonium is more common^3^.

In China, ammonium nitrogen contamination is a significant issue across river basins, primarily originating from the discharge of industrial wastewater, agricultural runoff, urban effluents, and leachate from landfills^4–6^. The concentration of total ammonium nitrogen in wastewater varies depending on the source. For example, ammonium concentrations in urban wastewater typically range from 10 to 200 mg·L⁻¹, while in industrial wastewater, concentrations can range from 5 to 1000 mg·L⁻¹^7^. Agricultural and livestock activities often release even higher levels of ammonium^8^. In view of the extensive and detrimental nature of ammonium nitrogen pollution^9–11^it is imperative to implement efficient wastewater treatment prior to discharge in order to reduce the ammonia nitrogen concentration.

Several physical and chemical methods are currently employed to address nitrogen pollutants in wastewater^12,13^. Despite their utility, these conventional methods are hindered by high operational costs, and inefficiency, and significant adverse environmental impacts^14,15^underscoring the urgent need for more sustainable alternatives. In contrast, biological nitrogen removal (BNR) technologies, driven by microorganisms, are gaining attention for their efficiency, cost-effectiveness, and lack of secondary pollution^16,17^. Among emerging solutions, microalgae-based nitrogen recovery technologies stand out for their ability to not only remove ammonia nitrogen efficiently but also to recover valuable resources, making them a sustainable and economically viable alternative to conventional methods^7,18–20^.

Microalgae are known to efficiently absorb ammonium nitrogen, typically preferring it over other nitrogen sources. However, at high concentrations or with prolonged exposure, ammonium nitrogen becomes toxic to algal cells, inhibiting their growth^7,20,21^. Although certain microalgae strains have been screened or acclimatized to tolerate elevated ammonium concentrations for wastewater treatment, these strains generally perform well only in wastewater with ammonium nitrogen concentrations below 500 mg·L⁻¹, or they require dilution or pre-treatment to support growth^7,22,23^. Ammonium toxicity remains a significant challenge in biological wastewater treatment. Therefore, mitigating ammonium stress in microalgae is critical for improving their pollutant removal efficiency in high-ammonium nitrogen wastewater.

Phytohormones play a vital role in regulating plant growth and enhancing stress resistance. The regulation of physiological and biochemical processes, as well as microalgal-bacterial interactions by phytohormones enhances microalgae’s resistance to various abiotic stresses^24–27^. For instance, phytohormones can upregulate antioxidant enzyme activities, improve photosynthetic efficiency, and modulate productivity^28–30^. Several studies have demonstrated that the exogenous application of phytohormones can alleviate nitrogen stress in microalgae cultures, improving their performance^31–33^. Additionally, research suggests that the crosstalk between nitrogen signaling and phytohormones in algae may be influenced by nitrogen-enriched or nitrogen-depleted growth media, which affects the endogenous concentration of these hormones^34,35^. Recent studies have also highlighted the potential of using phytohormones to improve nutrient and pollutant removal efficiency in wastewater treatment via microalgae^36–40^.

*Oocystis borgei* is a green alga isolated from a subtropical aquaculture pond of *Litopenaeus vannamei* by our laboratory^41^. Our previous research has demonstrated that *O. borgei* exhibits stable population dynamics^42^broad salinity tolerance^43^self-settling ability^44^and capacity for preservation at ambient temperature^45^demonstrating great potential for regulating aquaculture water and promoting shrimp growth and health^46^. Recent findings reveal that low concentrations (10⁻⁶ M) phytohormones, including indole-3-acetic acid (IAA), zeatin (ZT), and gibberellin A3 (GA3), effectively stimulate its growth and enhance nutrient removal (nitrogen and phosphorus) from seawater aquaculture wastewater^37^. While *O. borgei* preferentially utilizes ammonium nitrogen as its primary nitrogen source^47^the synergistic effects of phytohormones in enhancing its resistance to high ammonium nitrogen stress (HANS) remain underexplored, hindering the full application of this alga in large-scale wastewater treatment. Addressing this gap is crucial for maximizing the effectiveness of microalgae in ammonia nitrogen removal.

This study seeks to investigate how exogenous phytohormones can be leveraged to enhance *O. borgei*’s tolerance to HANS, ultimately improving its capacity for efficient and sustainable ammonia nitrogen removal. Specifically, low concentrations (10⁻⁶ M) of phytohormones (IAA, ZT, GA3) were supplemented under HANS conditions, and then growth, photosynthetic activity, antioxidant capacity, and nitrogen metabolism were measured to reveal the toxicity of HANS and the influence of phytohormones on the stress resistance of *O. borgei*. By elucidating these mechanisms, this research will pave the way for enhanced microalgae-based ammonia nitrogen removal systems, offering a sustainable and scalable solution to one of the most critical environmental challenges worldwide.

## Materials and methods

### Preculture and starvation treatment of *O. borgei*

The *O. borgei* strain, supplied by our laboratory, was pre-cultured in 5 L of Zhanshui 107 − 13 medium^37^ (prepared with autoclaved artificial seawater^48^) at 25 °C for 14 days under continuous illumination (21 µmol·m⁻²·s⁻¹) in the culture room. Cells were then harvested by centrifugation (5,000 rpm, 10 min), washed with sterile artificial seawater (salinity 30), and starved in N-deficient medium for 48 h. Prior to experiments, cells were resuspended in fresh N-deficient medium at 1.04 × 10⁶ cells·mL⁻¹ (OD₆₈₀ = 0.26).

### Experimental design

Dose-response analysis was conducted to study the toxic effect of high ammonium nitrogen on *O. borgei*. NH_4_Cl stock solution (10^5^ mg-N L^−l^) was used to create five different NH_4_^+^-N concentrations gradients (50, 200, 500, 800 and 1000 mg-N·L^−1^). For the control, N-sufficient medium was used. The initial pH of the medium was adjusted to approximately 7.0 using NaOH or HCl, s at this pH NH₄⁺ is the predominant form of ammonia nitrogen^1,7^.

To investigate the effect of phytohormones on *O. borgei* under HANS, *O. borgei* was cultured in a medium containing 500 mg-N L⁻¹, which was selected based on preliminary toxicity experiments. Subsequently, indole-3-acetic acid (IAA), gibberellic acid (GA3), and zeatin (ZT) (supplied by Hefei Bomei Biotechnology Co., Ltd. Hefei, China) were added to the culture at a concentration of 10^−6^ M, as previously demonstrated to promote the growth of *O. borgei*^37^. The control group was inoculated with cells in standard Zhanshui 107 − 13 medium.

### Culture conditions and growth indicators measurement

Cultures (200 mL in 250 mL flasks) were maintained in biological triplicates and then incubated in a light incubator (PRX-350 C, Ningbo Prant Instrument Co., Ltd., Ningbo, China) at 25 °C with a light intensity of 45 µmol·m⁻²·s⁻¹ and a 12 h/12 h light/dark photocycle for 7 days, with manual shaking four times daily. Growth kinetics were monitored through daily optical density measurements (OD₆₈₀) and specific growth rate calculations^37^ over the 7-day experimental period.

### Photosynthetic performance analysis

Comprehensive photosynthetic analyses were conducted at two critical timepoints (Days 4 and 7). The concentrations of photosynthetic pigments, including chlorophyll a (Chl a), chlorophyll b (Chl b), and carotenoids (Car), were determined using the hot-ethanol extraction method followed by spectrophotometric analysis (UV-1900i, Shimadzu, Japan). The quantification was performed according to established equations (Eqs. 1–3)^49^. Chlorophyll fluorescence parameters, including the actual photochemical efficiency (φPSII) of photosystem II (PSII) and the maximum quantum yield of PSII (Fv/Fm), were measured with a pulse-modulated fluorometer (FMS-2, Hansatech Instruments, Pentney, UK) with 594 nm amber modulation beam according to established protocols^44^. For the Fv/Fm measurement, samples were incubated in darkness at room temperature for 20 min before measurement. The photosynthesis-related gene ribulose-bisphosphate carboxylase (*rbcL*) was quantified via real-time quantitative PCR (qPCR). Total RNA extraction, cDNA synthesis and qPCR reaction were carried out according to the previous research methods^50^. Ribosomal protein S27 (*RPS27*) was employed as the internal reference gene to quantify the relative expression levels of the target genes (Table 1). The fold-change in gene expression was calculated using the comparative cycle threshold (2^*−ΔΔCt*^) method^51^.

1 $$\:\text{C}\text{h}\text{l}\text{a}\:\left({\upmu\:}\text{g}\cdot{\text{c}\text{e}\text{l}\text{l}}^{-1}\right)=\frac{\left(13.95{\:\text{O}\text{D}}_{665}-6.88\:{\text{O}\text{D}}_{650}\right)\times\:{\text{V}}_{\text{t}}\times\:\text{n}}{{\text{V}}_{\text{q}}\times\:\text{C}}\:$$

2 $$\:\text{C}\text{h}\text{l}\text{b}\:\left({\upmu\:}\text{g}\cdot{\text{c}\text{e}\text{l}\text{l}}^{-1}\right)=\frac{\left(24.96{\:\text{O}\text{D}}_{650}-7.32\:{\text{O}\text{D}}_{665}\right)\times\:{\text{V}}_{\text{t}}\times\:\text{n}}{{\text{V}}_{\text{q}}\times\:\text{C}}\:$$

3 $$\:\text{C}\text{a}\text{r}\:\left({\upmu\:}\text{g}\cdot{\text{c}\text{e}\text{l}\text{l}}^{-1}\right)=\frac{\left(1000{\:\text{O}\text{D}}_{470}-2.05\:\text{C}\text{h}\text{l}\text{a}-114.8\:\text{C}\text{h}\text{l}\text{b}\right)\times\:{\text{V}}_{\text{t}}\times\:\text{n}}{209\times\:{\text{V}}_{\text{q}}\times\:\text{C}}\:\:$$

where V_t_ is the extract volume (mL), n is the dilution factor, V_q_ is the sample volume (mL), and C is the cell density of *O. borgei* (cells·mL^−1^). Each absorbance value was corrected by subtracting the absorbance value at 750 nm.

**Table 1:** Genes and primers used in the study.

Gene	Primer sequences (5’−3’)	Amplicon length (bp)	Tm (˚C)	PCR Efficiency (%)	Correlation (*R*^2^)
*0rbcL*	F: CGTGGCGAAGAACTTTATGG	215	60	101.5	0.9964
	R: TGTCCGACGCCTCCCTGTA				
*GS*	F: ATGTGCGACACCTACGAACCC	147	60	99.0	0.9944
	R: CTCCTGCTCAATGCCAAACC				
*RPS27*	F: CAGGGCTGCTTCAACATCAC	160	60	97.8	0.9993
	R: CAGTTACAAATCCATCTCAGTCGC				

### Oxidative stress, antioxidant enzyme activities evaluation

10 mL of microalgae samples at the same sampling intervals were collected for evaluation of oxidative stress. Lipid peroxidation was evaluated by measuring malondialdehyde (MDA) content via thiobarbituric acid (TBA) assay. Antioxidant enzyme activities, including superoxide dismutase (SOD) and catalase (CAT), were measured according to the water-soluble tetrazolium (WST-1) method and ammonium molybdate method respectively. The assays followed the protocols provided by commercial kits (Nanjing Jiancheng, Nanjing, China).

### Nitrogen metabolism assessment

Nitrogen metabolism was investigated through both enzymatic and molecular approaches. Glutamine synthetase (GS) activity was measured colorimetrically, while transcriptional abundance of the nitrogen metabolism-related gene glutamine synthetase (*GS*) was quantified via real-time quantitative PCR (qPCR) as described above.

### Statistical analysis

Experimental data are presented as the mean ± standard deviation (SD), with *n* = 3. Data analysis was performed using GraphPad Prism 9. One-way ANOVA followed by Tukey’s multiple comparisons test was employed to assess significant differences among different treatments at the same time. Statistically significant differences are denoted by different letters (*p* < 0.05). For comparisons between different time points within the same treatment, unpaired t-tests were applied, and significant differences are marked with asterisks (^*^ *p* ≤ 0.05, ^**^ *p* ≤ 0.01).

## Results

### Effect of high ammonium nitrogen stress on the growth of *O. borgei*

**Fig. 1:**
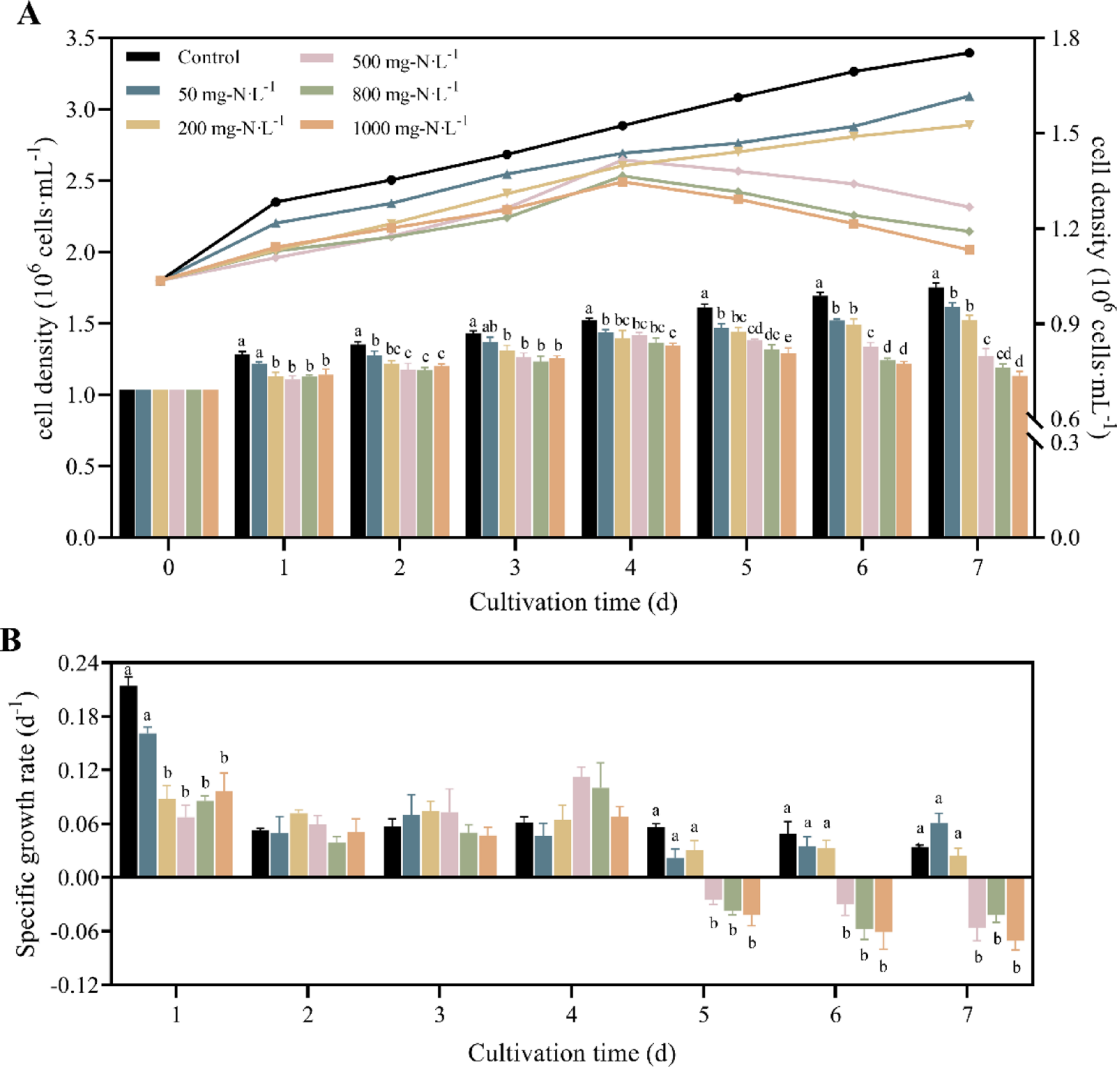
Effect of different concentrations of ammonium nitrogen on the growth of *O. borgei*. (**A**) Cell density; (**B**) Daily specific growth rate. Experimental data are presented as mean ± standard deviation (SD), with *n* = 3. Data analysis was performed using GraphPad Prism 9 with one-way ANOVA followed by Tukey’s multiple comparisons test. Statistically significant differences among treatments at the same time are denoted by different letters (*p* ≤ 0.05).

A dose-response analysis was conducted to determine the NH₄⁺-N concentration that induces stress in *O. borgei*. The results showed that NH₄⁺-N significantly inhibited *O. borgei* growth in a dose-dependent manner, with higher concentrations leading to more pronounced inhibition (Fig. 1). Specifically, after 7 days of exposure to NH₄⁺-N at concentrations of 50, 200, 500, 800, and 1000 mg·L⁻¹, cell density decreased by 7.77%, 12.98%, 27.70%, 32.08%, and 35.38%, respectively, compared to the control group (*p* ≤ 0.01). At lower NH₄⁺-N concentrations (50 and 200 mg·L⁻¹), cell density decreased compared to the control but positive growth trends were still observed (Fig. 1A). However, at 500 mg·L⁻¹ and above, cell density significantly decreased after 4 days of cultivation, resulting in negative growth (*p* ≤ 0.01), which persisted throughout the experiment (Fig. 1B). Based on these findings, 500 mg·L⁻¹ NH₄⁺-N was selected as the stress concentration for subsequent experiments.

### Effects of phytohormones on the growth of *O. borgei* under high ammonium nitrogen stress

**Fig. 2:**
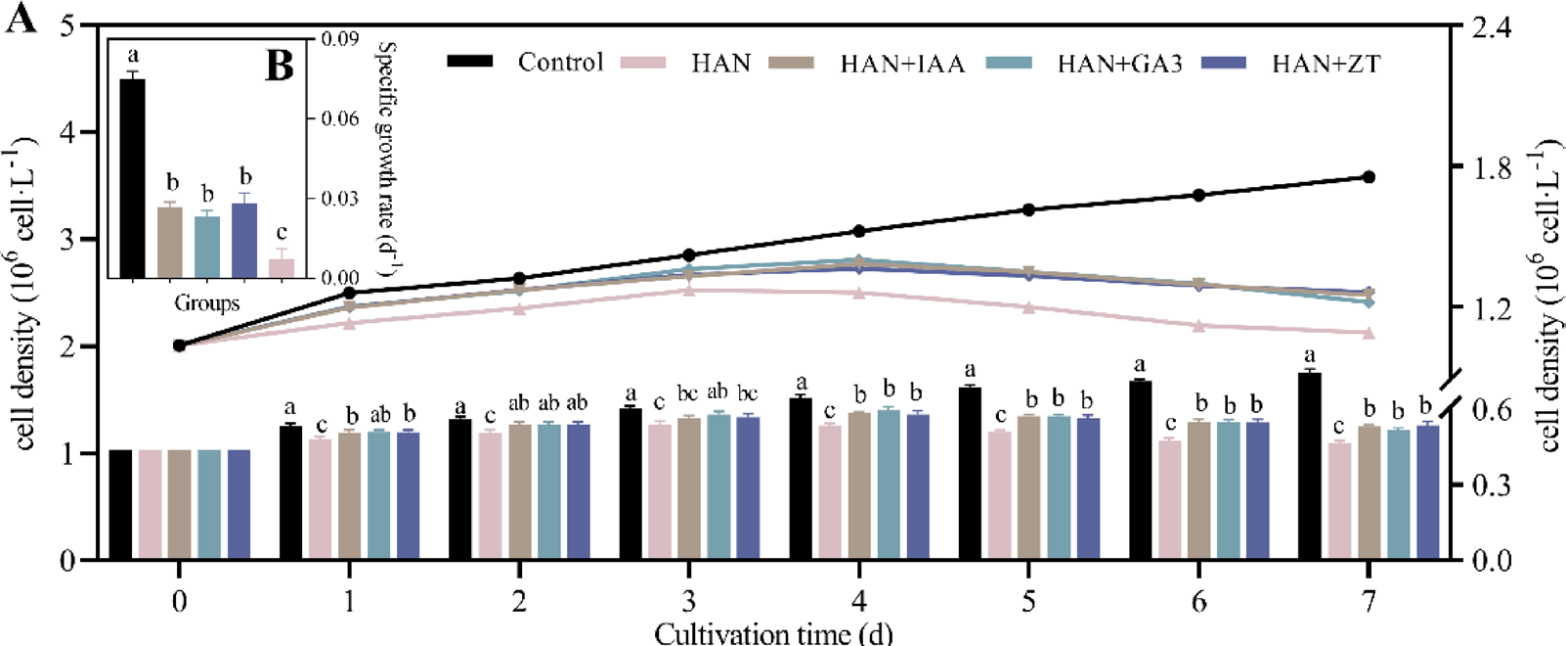
Effects of phytohormones on the growth of *O. borgei* under HANS. (**A**) Cell density; (**B**) Average specific growth rate over 7 d. HAN. High ammonium-nitrogen (500 mg-N L^−1^); HAN + IAA. High ammonium nitrogen with 10^−6^ M IAA; HAN + GA3. High ammonium nitrogen with 10^−6^ M GA3; HAN + ZT: High ammonium nitrogen with 10^−6^ M ZT. Experimental data are presented as mean ± standard deviation (SD), with *n* = 3. Data analysis was performed using GraphPad Prism 9 with one-way ANOVA followed by Tukey’s multiple comparisons test. Statistically significant differences among treatments at the same time are denoted by different letters (*p* ≤ 0.05).

The effects of phytohormones on *O. borgei* growth under HAN stress were evaluated by measuring cell density and specific growth rate (Fig. 2). The addition of 10⁻⁶ M IAA, GA3, and ZT significantly increased cell density compared to the HAN group (*p* ≤ 0.05), with the differences becoming highly significant after 4 days (*p* ≤ 0.01). After 7 days, cell densities in the IAA, GA₃, and ZT groups were 1.15, 1.12, and 1.16 times higher than those in the HAN group (*p* ≤ 0.01), respectively (Fig. 2A). The 7-day average specific growth rates for the IAA, GA3, and ZT groups were 0.027, 0.023, and 0.028 d⁻¹, respectively, significantly higher than the 0.007 d⁻¹ observed in the HAN group (*p* ≤ 0.001) (Fig. 2B). No significant differences were found among the three phytohormone treatments. These results demonstrate that phytohormones effectively alleviate the inhibitory effects of high NH₄⁺-N stress on *O. borgei* growth, highlighting their potential as growth regulators under stressful conditions. The 4th day emerged as a critical analytical timepoint, marked by negative growth in the HAN-treated *O. borgei* (Fig. 1) and significant cell density enhancement through IAA, GA3, and ZT supplementation (Fig. 2). So, physiological, biochemical, and molecular analyses were consequently conducted on days 4 and 7.

## **Phytohormone-mediated enhancement of stress tolerance in*O. borgei***

### **Alleviation of photosynthetic inhibition**

**Fig. 3:**
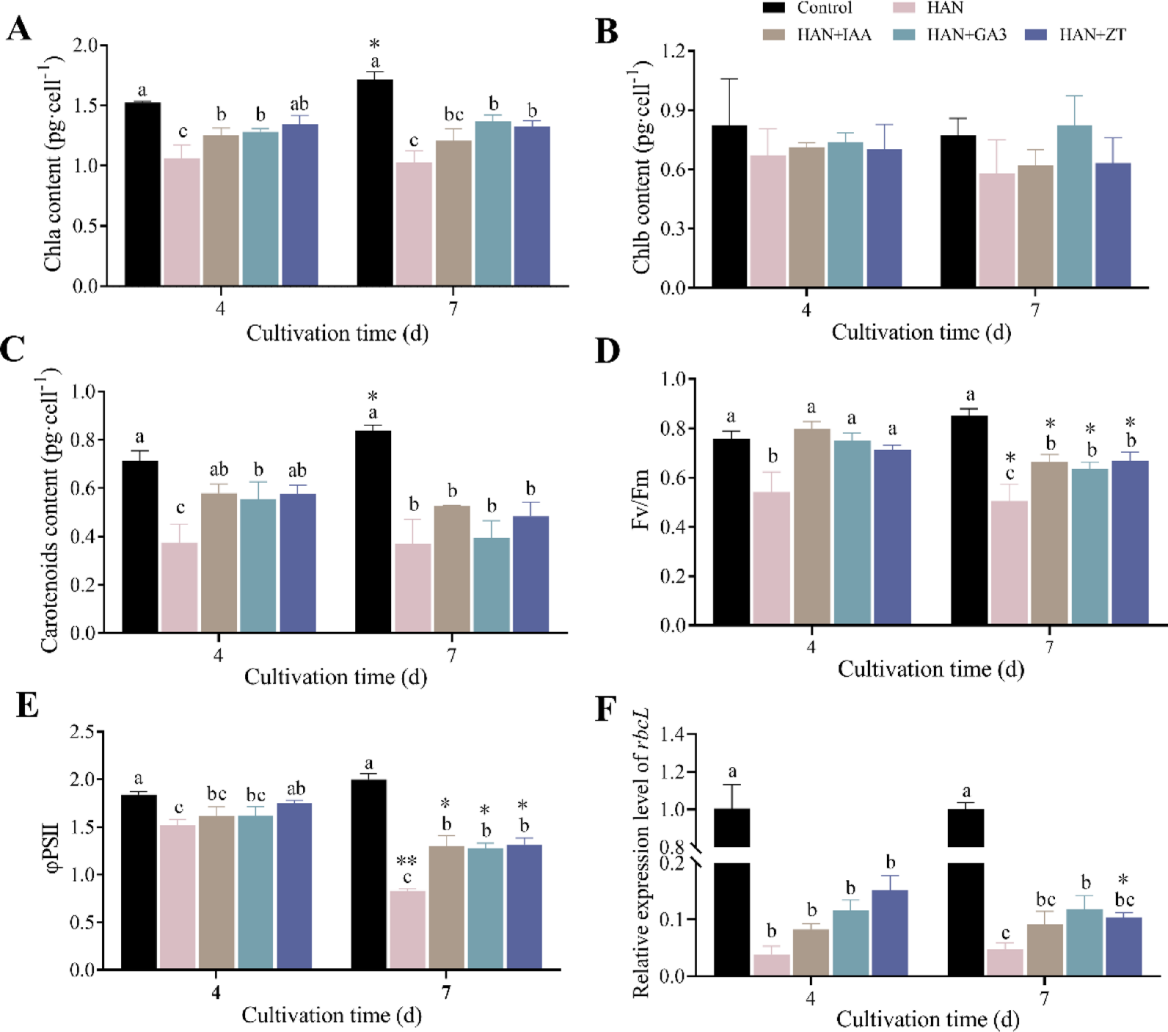
Effects of phytohormones on photosynthetic metabolism of *O. borgei* under HANS. (**A**) Chla content; (**B**) Chlb content; (**C**) Carotenoid content; (**D**) Fv/Fm; (**E**) φPSII; (**F**) relative expression level of *rbcL*. HAN. High ammonium-nitrogen (500 mg-N L^−1^); HAN + IAA. High ammonium nitrogen with 10^−6^ M IAA; HAN + GA3. High ammonium nitrogen with 10^−6^ M GA3; HAN + ZT: High ammonium nitrogen with 10^−6^ M ZT. Experimental data are presented as mean ± standard deviation (SD), with *n* = 3. Data analysis was performed using GraphPad Prism 9. One-way ANOVA followed by Tukey’s multiple comparisons test was employed to assess significant differences among different treatments at the same time. Statistically significant differences are denoted by different letters (*p* ≤ 0.05). For comparisons between different time points within the same treatment, unpaired t-tests were applied, and significant differences are marked with asterisks (* *p* ≤ 0.05, ** *p* ≤ 0.01).

To evaluate the impact of HAN stress on the photosynthetic activity of *O. borgei*, we measured the contents of Chla, Chlb, and Car (Fig. 3A-C). The contents of Chla and Car in the control group were significantly increased with time during cultivation (*p* ≤ 0.05). However, exposure to HAN stress caused a significant decline in Chla and Car contents. After 4 days, Chla and Car contents were reduced to 69.48% (*p* ≤ 0.0001) and 52.51% (*p* ≤ 0.001) of control levels, respectively, with no further significant decrease observed by day 7. In contrast, Chlb content remained relatively stable (*p* > 0.05). The supplementation of phytohormones (10⁻⁶ M IAA, GA₃, and ZT) effectively mitigated the decrease in Chla and Car contents. By day 4, Chla and Car levels in treated groups were 1.19–1.33 times (*p* ≤ 0.05) and 1.48–1.54 times (*p* ≤ 0.01) those of the HAN group, respectively. However, no significant differences were found among the three phytohormones (*p* > 0.05), and the alleviating effect of phytohormones on HAN stress did not exhibit a time-dependent pattern.

Additionally, we measured two chlorophyll fluorescence parameters (Fig. 3D and E) to assess the effects of phytohormones on photosynthetic activity. While no statistically significant differences were observed, the Fv/Fm and φPSII values in the control group gradually increased over time during cultivation (*p* > 0.05). In contrast, exposure to HAN stress resulted in a pronounced decline in both Fv/Fm and φPSII, with a significant downward trend observed over time. By day 7, these parameters had decreased to 59.3% (*p* ≤ 0.0001) and 41.6% (*p* ≤ 0.0001) compared to the control. Phytohormone treatment significantly alleviated photoinhibition under HAN stress. After 4 days, Fv/Fm under all phytohormone treatments and φPSII under ZT treatment recovered to levels that were not significantly different from the control (*p* > 0.05). Moreover, Fv/Fm and φPSII remained significantly higher than those in the HAN group after 7 days (*p* ≤ 0.01), despite a partial reduction in the alleviating effect.

We also examined the expression of the *rbcL* gene (Fig. 3F), which encodes the large subunit of Rubisco. HAN stress significantly reduced *rbcL* expression to 3.78% (*p* ≤ 0.0001) and 4.75% (*p* ≤ 0.0001) of control levels after 4 and 7 days, respectively. Phytohormone treatment resulted in a moderate increase in *rbcL* expression, though differences compared to the HAN group were not significant (*p* > 0.05), indicating a mild enhancement of *rbcL* expression under stress conditions. In conclusion, 10⁻⁶ M IAA, GA₃, and ZT enhanced the photosynthetic activity of *O. borgei* by increasing photosynthetic pigment content and chlorophyll fluorescence parameters, suggesting their potential as important factors in promoting resistance to HANS.

#### Reducing lipid peroxidation and alleviating oxidative stress damage

**Fig. 4:**
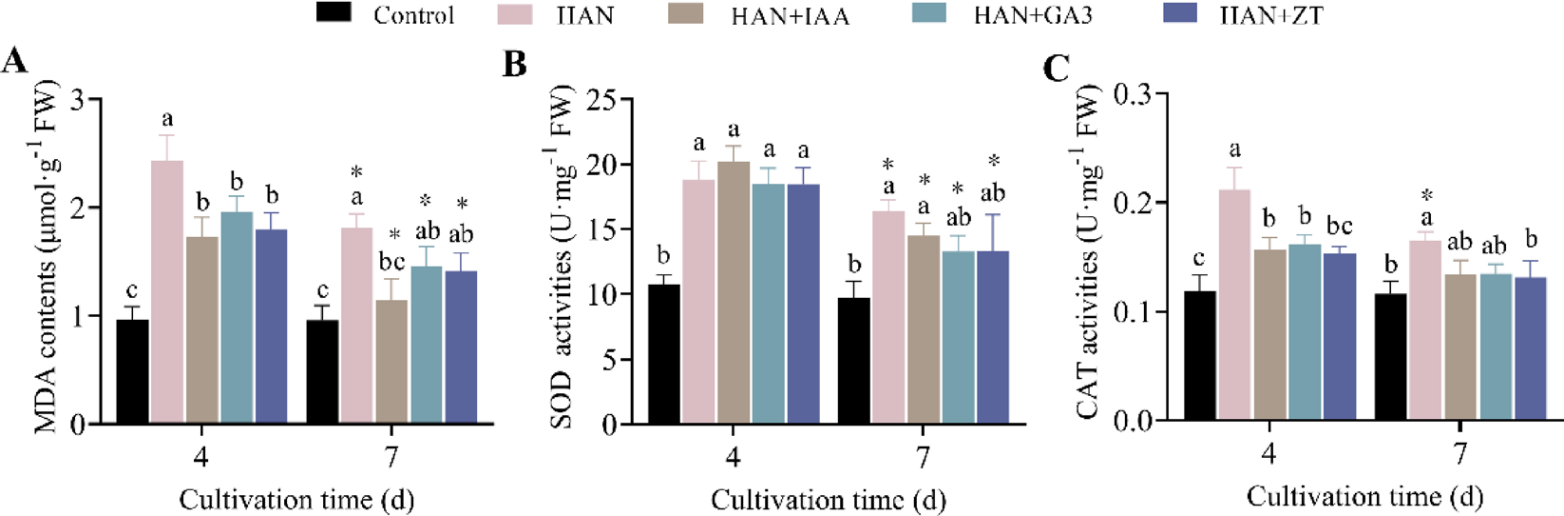
Effects of phytohormones on lipid peroxidation and antioxidant enzyme activities in *O. borgei* under HANS. (**A**) MDA content; (**B**) SOD activities; (**C**) CAT activities. HAN. High ammonium-nitrogen (500 mg-N L^−1^); HAN + IAA. High ammonium nitrogen with 10^−6^ M IAA; HAN + GA3. High ammonium nitrogen with 10^−6^ M GA3; HAN + ZT: High ammonium nitrogen with 10^−6^ M ZT. Experimental data are presented as mean ± standard deviation (SD), with *n* = 3. Data analysis was performed using GraphPad Prism 9. One-way ANOVA followed by Tukey’s multiple comparisons test was employed to assess significant differences among different treatments at the same time. Statistically significant differences are denoted by different letters (*p* ≤ 0.05). For comparisons between different time points within the same treatment, unpaired t-tests were applied, and significant differences are marked with asterisks (* *p* ≤ 0.05).

To explore the effects of HAN stress and phytohormones on oxidative stress and antioxidant systems in *O. borgei*, we measured malondialdehyde (MDA) content and the activities of catalase (CAT) and superoxide dismutase (SOD) (Fig. 4). Under HAN stress, MDA content, a marker of lipid peroxidation, significantly increased to 2.53 times the control level after 4 days (*p* ≤ 0.0001), then decreased to 1.89 times by day 7 (*p* ≤ 0.001) (Fig. 4A). This suggests an initial surge in oxidative stress followed by a partial recovery. SOD and CAT activities, key antioxidants, also increased sharply to 1.75 times (*p* ≤ 0.0001) and 1.79 times (*p* ≤ 0.001) the control levels at day 4, then slightly decreased to 1.43 times (*p* ≤ 0.001) and 1.68 times (*p* ≤ 0.01) by day 7 (Fig. 4B and C), indicating a persistent oxidative challenge. Phytohormone treatment significantly mitigated these oxidative stress indicators. At day 4, the increase in MDA content was substantially reduced in all phytohormone-treated groups compared to the HAN group. By day 7, MDA content in the HAN + IAA group decreased significantly (*p* ≤ 0.05) and approached control levels (Fig. 4A). Similarly, SOD activity in the HAN + GA3 and HAN + ZT groups and CAT activity in all phytohormone treatments returned to levels indistinguishable from the control group (*p* > 0.05) (Fig. 4B and C), indicating that these phytohormones effectively regulated antioxidant enzyme activities and reduced lipid peroxidation.

#### Promoting nitrogen metabolism in *O. borgei*

**Fig. 5:**
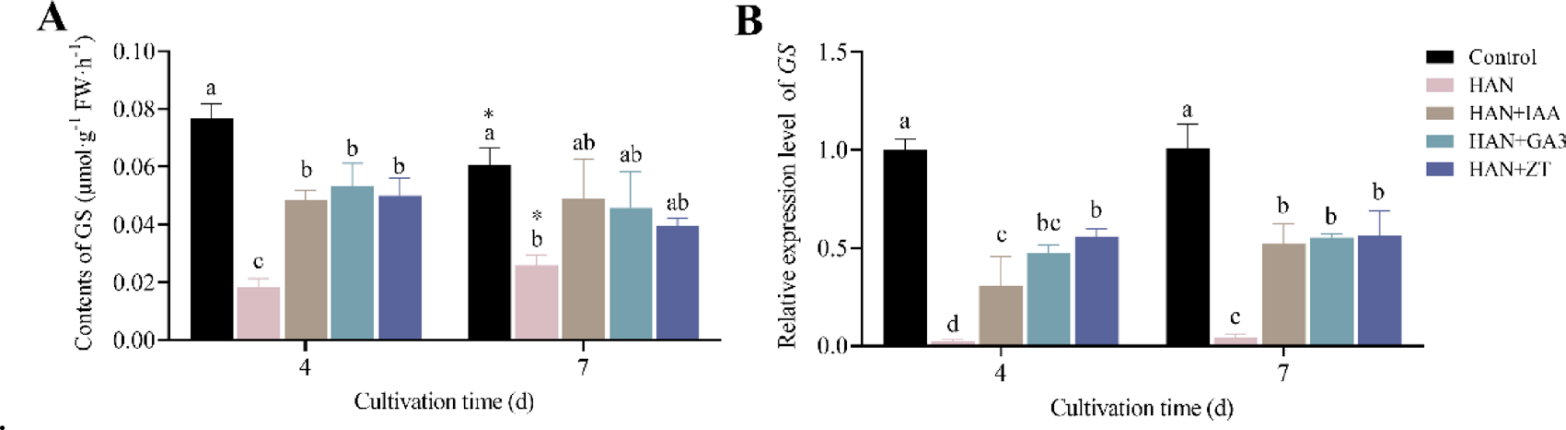
Effects of phytohormones on nitrogen metabolism in *O. borgei* under HANS. (**A**) Content of GS; (**B**) Relative expression level of *GS*; HAN. High ammonium-nitrogen (500 mg-N L^−1^); HAN + IAA. High ammonium nitrogen with 10^−6^ M IAA; HAN + GA3. High ammonium nitrogen with 10^−6^ M GA3; HAN + ZT: High ammonium nitrogen with 10^−6^ M ZT. Experimental data are presented as mean ± standard deviation (SD), with *n* = 3. Data analysis was conducted using GraphPad Prism 9. One-way ANOVA followed by Tukey’s multiple comparisons test was employed to assess significant differences among treatments at the same time. Statistically significant differences are denoted by different letters (*p* ≤ 0.05). For comparisons between different time points within the same treatment, unpaired t-tests were applied, and significant differences are marked with asterisks (* *p* ≤ 0.05).

To investigate the effects of HAN stress and phytohormones on nitrogen metabolism in *O. borgei*, we measured GS enzyme activity and *GS* gene expression levels (Fig. 5). Under HAN stress, there was a significant decrease in GS activity and gene expression over the 7-day period. At 4 days GS activity reduced to 23.91% of the control level (*p* ≤ 0.0001) and by 7 days, it further decreased to 42.86% (*p* ≤ 0.01) (Fig. 5 A). Similarly, GS gene expression was significantly reduced to 2.5% at 4 days and slightly increased to 4.22% at 7 days (*p* ≤ 0.0001) (Fig. 5B), indicating severe and persistent inhibition of nitrogen metabolism by HAN stress. Phytohormone treatments significantly enhanced nitrogen metabolism under HAN stress. At 4 days, GS activity increased significantly in all treated groups compared to the HAN group (*p* ≤ 0.001), with IAA, GA3, and ZT enhancing activity to 2.64, 2.91, and 2.73 times that of the HAN group, respectively. (Fig. 5 A). *GS* gene expression also rose significantly, with ZT showing the most pronounced effect at 22.40 times the HAN group level (*p* ≤ 0.001), followed by GA3 and IAA (Fig. 5B). Furthermore, the promoting effect of phytohormones on the GS activity and GS gene expression persisted until day 7 (*p* > 0.05). However, there were no significant differences among the three phytohormone treatment groups.

## Discussion

### Effect of ammonium stress on growth

Nitrogen is a vital element for the growth, development, and reproduction of microalgae, as it plays a key role in the formation of in proteins, nucleic acids, chlorophyll, and other vital substances within these organisms^52,53^. Among various inorganic nitrogen sources, NH₄⁺-N is generally preferred by microalgae. However, when present in high concentrations, NH₄⁺-N can inhibit growth^7,54^. Additionally, the degree to which NH₄⁺-N affects microalgae growth varies significantly depending on the species^55,56^strain^57^ and cultivation conditions^20^. Metin et al. (2024) investigated the impact of varying NH₄⁺-N concentrations on the growth of four microalgae species: *Chlorella vulgaris*, *Chlorella minutissima*, *Chlamydomonas reinhardtii*, and *Arthrospira platensis*. Their findings indicated that *C. vulgaris* exhibited the highest resistance to ammonium toxicity, while *A. platensis* experienced significant growth inhibition. Notably, none of these species were able to thrive when NH₄⁺-N concentrations exceeded 1000 mg L^−1 21^. In our study, we observed that NH₄⁺-N inhibited the growth of *O. borgei* in a dose-dependent manner. Growth inhibition observed starting from day 4 of cultivation when NH₄⁺-N concentration was ≥ 500 mg L^−1^ (Fig. 1). This finding consistent with the results of Zhao et al. (2019), who reported that ammonium nitrogen concentrations above 500 mg L^−1^ were highly toxic to *T. cordiformis*^33^. The similarity of these findings across different microalgae species emphasizes the importance of carefully managing ammonium nitrogen levels in microalgae cultivation systems.

### Effects of phytohormones on microalgae growth under abiotic stress

Phytohormones have emerged as effective regulators of microalgae growth under abiotic stress conditions, demonstrating significant potential for enhancing stress tolerance and biomass production^25,58^. In our study, the application of phytohormones (10^−6^ M IAA, GA3, ZT) alleviated the negative effects of high ammonium stress, resulting in a 12–16% increase in cell density and an 85–131% increase in the specific growth rate (Fig. 2). These findings are consistent with previous studies on other microalgae species under various stress conditions. For instance, exogenous melatonin (MT) and indole-propionic acid (IPA) have been shown to enhance biomass and carbon fixation capacity in *Spirulina platensis* under low light conditions^59^. Similarly, the exogenous application of phytohormones such as IAA, ZT, and brassinolide (Br) alleviated oxidative damage and photosynthetic inhibition in *T. cordiformis* under high ammonia nitrogen stress^33^. Another study found that supplementing *Scenedesmus* SDEC-8 and *Chlorella* SDEC-18 with indole-3-butyric Acid (IBA) and naphthalene acetic acid (NAA) maintained biomass concentration, mitigated nitrogen stress damage, and increased lipid yield in microalgae cells^60^. Other studies have also observed that the exogenous application of phytohormones like Br, GAs, auxins, cytokinins (CTKs), and MT, or synthetic phytohormones, can effectively promote microalgae growth by mitigating the adverse effects of abiotic stress factors such as heavy metals, high/low temperatures or light, high salinity, and nitrogen deficiency^30–32,61–63^. These findings support our results and highlight the broad applicability of phytohormone treatments in enhancing microalgae stress tolerance.

It should be noted that different phytohormones exhibit varying degrees of effectiveness in responding to abiotic stress, primarily due to their distinct characteristics. For instance, auxins and CTKs promote cell division and elongation, GAs enhance overall metabolic activity and stress resistance, while abscisic acid (ABA) regulates stress responses and carbon allocation in microalgae^25,39,58,64^. This variation highlights the necessity of selecting and optimizing phytohormones when culturing algae and managing stress. However, in our study, the three phytohormones did not show significant differences in cell density of *O. borgei* under HAN stress, which suggests that cost-effectiveness should be considered before the large-scale application^37^.

### Phytohormones alleviate photosynthetic Inhibition

Photosynthesis is a fundamental process for the growth and development of plants, and abiotic stress primarily reduces photosynthetic efficiency^65^. Similarly, our study shows that HANS (500 mg L^−1^ NH₄⁺-N) causes photosynthetic damage in *O. borgei*. This damage is reflected in reduced photosynthetic pigment content (Chla and Car), decreased chlorophyll fluorescence parameters (Fv/Fm and φPSII), and lowered expression of the photosynthetic gene *rbcL* (*p* ≤ 0.01) (Fig. 3).

Under abiotic stress conditions, phytohormones have become critical regulators of photosynthetic efficiency in microalgae. For example, phytohormones such as auxins, CTKs, ABA, Br, and MT have been shown to promote the synthesis of chlorophyll and carotenoids, essential for light absorption and energy transfer^25,33,59^. This enhancement in pigment content directly contributes to improved photosynthetic efficiency by increasing the light-harvesting capacity^66,67^. In our study, we observed a significant increase in chlorophyll and carotenoid levels following phytohormone treatment (*p* ≤ 0.01) (Fig. 3A-C), which indicates enhanced light-harvesting efficiency and energy transfer in *O. borgei*.

Furthermore, phytohormones can enhance light energy conversion efficiency and the activity of the photosystem reaction centers, promoting photosynthetic metabolic responses in *O. borgei*. This is evidenced by increased ratios of Fv/Fm and φPSII under stress conditions (Fig. 3D and E). Fv/Fm is an indicator of the energy capture efficiency of PSII, a higher value indicates greater potential for light energy utilization. φPSII reflects the proportion of absorbed light that is used in PSII photochemistry^68^. Our previous research has also shown that 10^−6^ M IAA, GA3 and ZT can significantly enhance Fv/Fm and φPSII values in *O. borgei* under high-salt wastewater conditions^37^. Similarly, phytohormone supplementation has been shown to increase Fv/Fm in *Chlorella sorokiniana* under nitrogen-limiting conditions^69^ and *T. cordiformis* under HANS^33^.

The *rbcL* gene encodes the large subunit of Rubisco, a key enzyme for carbon assimilation, that catalyzes the primary reaction of carbon fixation and is crucial for photosynthesis^70^. Changes in *rbcL* expression under stress conditions reflect the extent of photosynthetic impairment^32,71^. In our study, *rbcL* expression was significantly inhibited under HANS (*p* ≤ 0.0001). Despite the improvement in photosynthetic efficiency following phytohormone supplementation, *rbcL* expression did not recover significantly (Fig. 3F). This discrepancy suggests that enhanced photosynthetic efficiency does not solely depend on the recovery of *rbcL* expression. Previous studies have shown that phytohormones can partially compensate for the lack of Rubisco activity by protecting essential photosynthetic proteins, increasing the content of photosynthetic pigments, and optimizing the photosynthetic electron transport chain^72,73^. Moreover, phytohormones can indirectly improve photosynthetic efficiency by activating the antioxidant defense system and reducing ROS-induced damage to the photosynthetic system^74^. This regulatory mechanism may offset the impact of insufficient *rbcL* expression on photosynthetic efficiency, explaining the observed improvement in photosynthetic parameters despite the lack of significant recovery in *rbcL* expression.

### Phytohormones mitigate oxidative stress

The redox balance plays a crucial role in innate immunity, a feature universally present across all algal phyla. Under normal growth conditions, microalgae produce ROS, which activate antioxidant systems to maintain intracellular redox balance^75^. However, when abiotic stress persists over extended periods, ROS production can exceed the capacity of the cell’s antioxidant removal systems, leading to oxidative stress. Excess ROS can react with unsaturated fatty acids in the lipid cell membrane, producing MDA, a byproduct of lipid peroxidation. This disrupts cellular function and can eventually result in cell death^74,76^.

In response to abiotic stress, microalgae mitigate oxidative damage through both enzymatic and non-enzymatic antioxidant systems. Key antioxidant enzymes, such as SOD and CAT, are essential for microalgal cells and are commonly used as physiological indicators of stress response^75^. Typically, prolonged exposure to unfavorable conditions prompts microalgae to upregulate antioxidant levels, facilitating adaptation to long-term abiotic stress^74–76^. In this study, we observed that HAN stress induced lipid peroxidation and oxidative stress in *O. borgei*, with significantly elevated levels of MDA, SOD, and CAT compared to the control group (*p* ≤ 0.001). In contrast, HAN stress on *T. cordiformis* caused damage to its enzymatic antioxidant system^33^suggesting that *O. borgei* exhibits stronger redox regulation capabilities.

Previous reports have indicated that plant hormone signaling is closely linked to ROS regulation^30,77^. Exogenous phytohormones can act as antioxidants, regulating the antioxidant system to prevent ROS accumulation, and thereby limiting or alleviating oxidative damage caused by abiotic stress^39,74,78^. In our study, phytohormones significantly enhanced carotenoid content under HAN stress early in the treatment period (*p* ≤ 0.5) (Fig. 3C), while MDA, SOD, and CAT levels were significantly reduced throughout the entire culture period (*p* ≤ 0.05) (Fig. 4). Carotenoids, as non-enzymatic antioxidants, scavenge free radicals, particularly superoxide anions and hydrogen peroxide radicals^79^. Under abiotic stress, the promotion of carotenoid synthesis via exogenous phytohormones is a a strategy employed by *O. borgei* to combat ROS toxicity, reducing lipid peroxidation caused by HAN and alleviating pressure on the enzymatic antioxidant system. Similarly, exogenous auxins, CTKs, and MT help maintain redox balance in microalgae by regulating antioxidant systems or related gene expression under environmental stress^73,78,80^.

This study also found that different phytohormones varied in their effectiveness at alleviating oxidative stress caused by HAN. All three plant hormones positively regulated CAT activity, with IAA showing the most significant effect in reducing lipid peroxidation damage. Although IAA led to higher SOD activity, the SOD activity under GA3 and ZT treatments did not differ significantly from the control group. A similar phenomenon was observed in the alleviation of lead toxicity in *Acutodesmus obliquus* by phytohormones, where IAA resulted in higher SOD activity, while tZ (trans-zeatin) showed higher CAT activity^73^. The differences in the effects of phytohormones on antioxidant enzyme regulation are likely result from their distinct roles in cellular signaling and metabolic pathways^39^.

### Phytohormones enhance nitrogen metabolism

The role of phytohormones in regulating nitrogen metabolism under environmental stress has been extensively studied in higher plants^28,81^yet research on how phytohormones alleviate environmental stress in microalgae, particularly concerning nitrogen metabolism remains limited^33^. This study contributes valuable insights into this area, focusing on the effects of phytohormones on nitrogen metabolism in *O. borgei* under HAN stress. During nitrogen metabolism, GS is a critical enzyme not only for the assimilation of NH_4_^+^ but also for plant growth and development^82^. Under HAN stress, we observed a significant downregulation in GS enzyme activity and near-complete suppression of *GS* expression (*p* ≤ 0.0001) (Fig. 5), indicating that nitrogen metabolism in *O. borgei* is impaired. This dysfunction in nitrogen metabolism is a primary factor contributing to the inhibitory effects of 500 mg L^−1^ NH_4_^+^-N on microalgal growth. Importantly, our results show that the phytohormones IAA, GA3, and ZT significantly increased GS activity (*p* ≤ 0.001) and upregulated *GS* expression (*p* ≤ 0.05) in *O. borgei* under HAN stress (Fig. 5). These findings are consistent with recent studies on other microalgal species. For example, Zhao et al. (2019) reported that Br enhanced nitrogen assimilation in *T. cordiformis* under high ammonia nitrogen stress by increasing GS activity and gene expression^33^.

Enhancing nitrogen metabolism is a potential strategy for mitigating damage caused by external stresses. GS plays a central role in nitrogen assimilation and amino acid biosynthesis. Its enhanced activity facilitates more efficient ammonium incorporation and detoxification, which is particularly critical under HAN stress in this study. Moreover, improved nitrogen metabolism might contribute to the synthesis of stress-protective compounds such as proline and glycine betaine, further enhancing the overall stress resistance of microalgae^83,84^. By promoting GS activity and gene expression, phytohormones may enable more efficient nitrogen assimilation and utilization, thereby mitigating the toxic effects of high ammonium concentrations. This enhanced nitrogen metabolism likely plays a key role in the observed improvements in growth and stress tolerance in *O. borgei* under high ammonium conditions.

### Study limitations and future directions

While this study advances understanding of phytohormone-mediated enhancement in *O. borgei* tolerance to high ammonium stress, it has limitations including a sole focus on *O. borgei* monocultures (neglecting microbial community influences), incomplete elucidation of the underlying molecular mechanisms, lack of validation under realistic wastewater conditions, and investigation of only a limited subset of phytohormones. Future research should therefore focus on characterizing microbial community dynamics and *O. borgei*-bacteria interactions, employing transcriptomic and metabolomic analyses to identify key regulatory genes and pathways, conducting pilot-scale or field trials using actual wastewater, and exploring a broader spectrum of phytohormones for efficacy. Addressing these aspects will yield a more comprehensive understanding and advance the development of efficient microalgae-based wastewater treatment technologies.

## Conclusion

This study demonstrates that NH_4_^+^-N significantly inhibits *O. borgei* growth in a dose-dependent manner, with 500 mg L^−1^ NH_4_^+^-N (HAN) causing growth inhibition and physiological metabolic disturbances. Phytohormones (10^−6^ M IAA, GA3, and ZT) significantly enhanced the tolerance of *O. borgei* to HAN stress. Specifically, these phytohormones increased pigment content, optimized electron transport, and enhanced photosynthetic efficiency. They also mitigated oxidative damage by regulating antioxidant defense mechanisms and boosted nitrogen metabolism by promoting GS activity and gene expression. As a result, these treatments resulted in a 22–26% increase in cell density and a 217–282% increase in the specific growth rate. These findings highlight the potential of phytohormones as effective tools for enhancing microalgal tolerance to NH₄⁺-N stress for high ammonium-nitrogen wastewater treatment. For practical implementation, we propose optimizing hormone delivery protocols (e.g., timing, dosage, and combinatorial ratios) in cultivation systems and conducting species-specific trials to evaluate the broader applicability of this approach in microalgae-based wastewater remediation and biomass production.

## Data Availability

The authors will make the raw data supporting the conclusions of this article available without undue reservation. The data is available from the corresponding author on reasonable request. (Ning Zhang: zning496@163.com)
